# Novel non-invasive in-house fabricated wearable system with a hybrid algorithm for fetal movement recognition

**DOI:** 10.1371/journal.pone.0254560

**Published:** 2021-07-13

**Authors:** Upekha Delay, Thoshara Nawarathne, Sajan Dissanayake, Samitha Gunarathne, Thanushi Withanage, Roshan Godaliyadda, Chathura Rathnayake, Parakrama Ekanayake, Janaka Wijayakulasooriya

**Affiliations:** 1 Department of Electrical and Electronic Engineering, Faculty of Engineering, University of Peradeniya, Peradeniya, Sri Lanka; 2 Department of Obstetrics and Gynacology, Faculty of Medicine, University of Peradeniya, Peradeniya, Sri Lanka; Fuzhou University, CHINA

## Abstract

Fetal movement count monitoring is one of the most commonly used methods of assessing fetal well-being. While few methods are available to monitor fetal movements, they consist of several adverse qualities such as unreliability as well as the inability to be conducted in a non-clinical setting. Therefore, this research was conducted to design a complete system that will enable pregnant mothers to monitor fetal movement at home. This system consists of a non-invasive, non-transmitting sensor unit that can be fabricated at a low cost. An accelerometer was utilized as the primary sensor and a micro-controller based circuit was implemented. Clinical testing was conducted utilizing this sensor unit. Two phases of clinical testing procedures were done and during the first phase readings from 120 mothers were taken while during the second phase readings from 15 mothers were taken. Validation was done by conducting an abdominal ultrasound scan which was utilized as the ground truth during the second phase of the clinical testing procedure. A clinical survey was also conducted in parallel with clinical testings in order to improve the sensor unit as well as to improve the final system. Four different signal processing algorithms were implemented on the data set and the performance of each was compared with each other. Out of the four algorithms three algorithms were able to obtain a true positive rate around 85%. However, the best algorithm was selected on the basis of minimizing the false positive rate. Consequently, the most feasible as well as the best performing algorithm was determined and it was utilized in the final system. This algorithm have a true positive rate of 86% and a false positive rate of 7% Furthermore, a mobile application was also developed to be used with the sensor unit by pregnant mothers. Finally, a complete end to end method to monitor fetal movement in a non-clinical setting was presented by the proposed system.

## Introduction

During pregnancy, the main aim of the parents, as well as the obstetricians, is to maintain the health of the fetus as well as the mother. Obstetricians use different methods to assess fetal health. Among them monitoring fetal movement is the most commonly used method. It is also the simplest and the most economical method available [1].

Several studies have been carried out to classify the different types of fetal movements [2]. It was mentioned that fetal movements can be classified based on the amplitude and the speed of the specific activity. The amplitude could be weak vs strong and the length of the activity could be short vs sustained. In a study conducted [3] seven different fetal movement types were identified using Ultrasound imaging and Doppler Ultrasound. They were Startles, General movements, Hiccups, Fetal breathing movements, Isolated arm or leg movement, Twitches and Clonic movements. It was also noted that there is a striking similarity between the observed movements and the movements observed in a baby after birth. However, this research focuses only on identifying the occurrence of a fetal movement utilizing an acelerometric sensor as oppose to classifying different types of fetal movements.

In a survey conducted 99.9% of pregnant mothers reported that it was important to feel and count baby movements [4]. Fetal movement patterns of each trimester of the pregnancy may differ from each other as well as from each fetus and mother. Studies have shown that the perception of decreased fetal movement is associated with stillbirth [5]. Any difference in the fetal movement pattern can indicate that the fetus is unwell. The change in the pattern could be reduced fetal movement, weak fetal movement or intense fetal movement. Hence, change in fetal movement pattern may be a sign of an unhealthy fetus [3]. It was also shown that by timely reporting to health care providers when experiencing a decreased fetal movement may prevent perinatal morbidity and mortality [2]. A study carried out on 305 women who experienced reduced fetal movement after 28 weeks of gestation showed that 22.1% pregnancies ended in complications such as small-for-gestational-age infants [6]. Another study reported 54.7% cases of stillbirth resulting women present who experienced reduced fetal movements [7]. In another study 20 out of 23 growth-restricted fetuses were identified prior to birth by monitoring fetal movement counting where as only 12 out of 20 growth-restricted fetuses were identified with standard antenatal care without fetal movement counting [2]. Also, several studies have been conducted to identify fetal movement patterns in each trimester as well as fetal movement patterns in of mothers [2, 6, 7]. Another study conducted by the Royal College of Obstetricians and Gynecologists has highlighted the lack of studies of fetal movement patterns [8]. Therefore, it can be concluded that monitoring fetal movement patterns play a major role in fetal well-being. Furthermore, further studies need to be conducted on how fetal movement patterns effect the well-being of the fetus.

Currently, fetal movements can be quantified by conducting an ultrasound scan or an MRI scan [9, 10]. These can only be conducted in a clinical setup and can only be done by a trained technician [9]. In a study, an automated method of analysing fetal growth using 2D ultrasound was developed [10]. This was done due to the lack of trained sonographers in developing countries. This indicates that there’s a lack of trained technicians to conduct these tests. As a result, these tests are expensive and can only be conducted in a short window of time.

Few research studies were conducted on monitoring fetal movement via non-invasive techniques. A study conducted using a single accelerometer placed on the mother’s abdomen employed a threshold signal processing method [11]. This study concluded that the thresholding method employed to identify fetal movement performed poorly. Also, it was found that the acceleration signals are corrupted by maternal movements such as laugh, cough and hiccup. Therefore, a more complex signal analysis method needs to be employed. Another study [12] employed a capacitive accelerometer to monitor fetal movements while the mother was asleep. In the initial part of the experiment, an ultrasonographer was used parallel to the device to validate data acquired. During this experiment, three types of fetal movements were recorded and their positive hit rates were calculated. Gross movements had a positive hit rate of 38.5%—23.5% depending on the fetal age. Similarly, isolated limb movements had a positive hit rate of 5% to 13% and breathing movements had a lower positive hit rate of 4.3%—22.8%. Another study [13] was conducted using acoustic sensors and accelerometer sensors. This was also conducted in a clinical setup with ultrasound validation. They were able to discriminate fetal startle movements from general movements with 72.1% accuracy. Several other research studies also have been conducted on this topic [14–16].

In this study, we investigate whether an accelerometer sensor can be used to monitor the fetal movement count in a non clinical setting.

## Materials and methods

### Hardware

Few studies were conducted on wearable sensor-based fetal monitoring devices [11, 12, 17]. The most common sensors used in previous studies were accelerometers and acoustic sensors. Furthermore, it was mentioned in a study conducted that it is possible to monitor fetal movements as well as heartbeat using an accelerometric sensor [18]. Therefore, it was decided that an accelerometric sensor should be used to capture data. However, it was also mentioned in the study [18] that this is realizable only after the 30th week of gestation. This is due to the lack of strength of the signals generated when the fetus is in the early gestational stages. However, this proposed system was able to identify the fetal movement of fetuses from the gestation age of 26 weeks onwards utilizing the accelerometric sensor.

A device was initially developed to acquire signals from pregnant mothers. When selecting a sensor for this device several factors were considered: One of the main considerations made was using a non-invasive sensor to acquire data. This was to reduce the impact of the device on the fetus as well as on the mother. Furthermore, the main objective of this was to come up with a wearable device which can be used by mothers daily. Therefore the ergonomics of the sensor also played a major role. The sensor to be selected should be light in weight and small in size. Also, it should be easy to wear on the mother’s abdomen. Since this device should be commercially sold the cost also play a major role.

Considering all these factors our research team decided to use the sensor MPU 9250. It is a multi-chip module which houses a 3-Axis accelerometer and a 3-Axis gyroscope. It has inbuilt analogue to digital converters to convert the signals received from the accelerometer and the gyroscope. The data received from the sensor is transferred to a removable micro SD card via a microcontroller. This enables the device to operate independently of a computer and also eliminates the need for a wireless transfer method which may have adversary effects on the fetus [17]. The microcontroller unit utilized in the device is the Microchip ATmega328P microcontroller. This is embedded in an Arduino Uno board developed by Arduino.cc. This chip is a high performance low-power CMOS 8-bit microcontroller with the read-while-write capability.

A study was conducted using accelerometers to decide the optimum number of sensors to be used and the sensor positioning [19]. In this study, the number of sensors was varied from one to five and the positive predictive value of each instance was observed. The increase in the number of sensors was found not to have a considerable effect on the positive predictive value according to this study. Therefore, it was decided that a single sensor would be sufficient for this setup (for fetal movement detection). This idea was further supported by the notion that this will reduce the computational capacity required as well as the size of the data recorded which in turn will improve the speed of analysis as well as data transfer.

The device was powered utilizing a standard power bank. The sensor and the microchip both operate at low power. Therefore, the capacity of a standard power bank is more than sufficient for a session. However, the power bank should be selected such that the maximum supply voltage to be 5v, maximum current supply to be 1A and the minimum leakage current to be less than 100mA. The actual duration of operation depend on the capacity of the used power bank.

**Fig 1:**
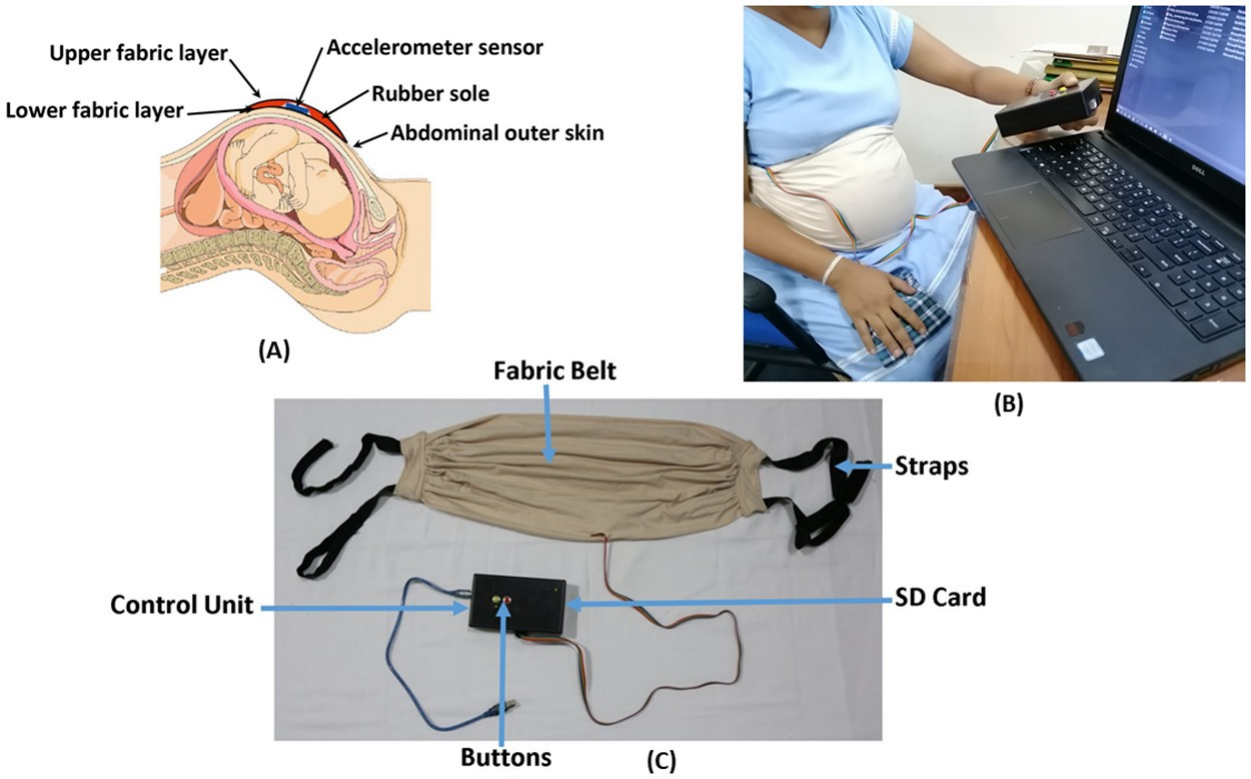
The in-house designed and fabricated device. (A)The illustration of the cross section of the deice. (B) A photo taken during clinical testing. the belt is secured to mothers abdomen and the button system is being used to record maternal perception (C)All the components of the device: the fabric belt, the sensor, the micro SD card and the control box are clearly labeled.

The clinical testing procedure was conducted in two phases. During the initial phase mothers perception of fetal movement was considered to be the ground truth. However, during the second phase, ultrasound readings were utilized for validation and as the ground truth. During both of these phases, a belt-like device was used to collect data. This can be seen in Fig 1.

Initially the sensor, MPU 9250 was embedded into a rubber sole. This was to adhere the sensor on to the abdomen securely as well as to prevent any discomfort to the mother due to the sharp edges of the sensor. Then this rubber sole was stitched into a fabric belt. The choice of the material to the fabric belt was also done considering the mother’s comfort. Several factors such as the materials ability to absorb perspiration, flexibility, how well it moulds to the mother’s abdomen as well as the colour of the material were considered when choosing the material. This selection was made by considering the responses of several pregnant mothers who were interviewed during the design process. The dimensions of this belt were designed in such a way so that it could be worn for an extended time period. Furthermore, the sensor was placed at the centre of the belt in order to obtain a uniform sensor positioning on every mother. The sampling rate was set to 280 samples per second and acceleration measurements were recorded. The amplitude of the acceleration was measured in Accelerometric Measurements units (AMU).

During the clinical testing period, the micro controller as well as the SD card were housed in a separate box. Two buttons were also included in this setup. This housing can be seen in Fig 1. One of the buttons was used to obtain mothers perception of fetal movements. The other button was used to record maternal movements such as laugh, cough and hiccups. During clinical testing, the mother was advised to press Button 1 when a fetal movement was felt. The occurrence of maternal laugh was marked by the research group by pressing Button 2. The information from these buttons are retrieved by the microcontroller and recorded alongside the accelerometric sensor data. Therefore, this data was used for the labelling of classes.

#### Ethical clearance

This study was approved by the Ethics Review Committee, Faculty of Medicine, University of Peradeniya. Approval was granted to conduct research project No. 2018/EC/43 entitled “Fetal movement analysis for condition monitoring” at the Teaching Hospital, Peradeniya.

### Clinical tests

The clinical testing procedure was conducted in two phases. During phase one mother’s perception of fetal movements were considered to be the ground truth. During this phase, the design and development of the device were also done. Some adjustments were made to the device during this period according to the feedback received from pregnant mothers. At the conclusion of phase one, a proper device was designed and a proper method of taking readings was developed. Then at phase two ultrasound readings were considered to be the ground truth. The device which was finalized in phase one was utilized in this phase to obtain readings.

**Phase 1**: During this phase readings from 120 women impatient at Peradeniya Teaching hospital, Sri Lanka were taken. The gestational age of the group varied from 28 weeks to 40+ weeks and most were singleton pregnancies. A twin pregnancy as well as a quadruplets pregnancy was also recorded. However, only the readings from singleton pregnancies were initially considered. Furthermore, the occurrence of fetal movements and the occurrence of maternal movements were recorded utilizing the button system mentioned above.

**Phase 2**: During this phase readings from 15 mothers were taken and all of them were impatient at Peradeniya Teaching Hospital, Sri Lanka. Similar to phase one the gestation period of these mothers varied from 28 weeks to 40+ weeks and all the pregnancies were singleton. Furthermore, during this phase, each mother underwent an abdominal ultrasound, and the fetal movements were monitored and recorded by a trained technician while the developed device recorded accelerometric data. Other maternal movements were also recorded. Moreover, the mother’s perception was also recorded. This is done in order to have a proper comparison between maternal perception and the fabricated device. Currently, maternal perception of fetal movement counting is the method used by mothers. The proposed system will provide a low-cost less cumbersome alternative to this method. Hence during this phase, while ultrasound was utilized to establish the ground truth, the maternal perception was also considered and recorded in order to compare the performance of the existing method which is the maternal perception and the proposed new method which is the system introduced in this research.

When conduction the clinical tests, as the ethical clearance provided by the Ethics Review Committee, Faculty of Medicine, University of Peradeniya allows, all the participants were recruited from the Teaching Hospital, Peradeniya. The selection process of the participants was done randomly, where, their physique, as well as the age, were disregarded. However, participants with a gestation age between 26 weeks to 40+ weeks were selected, due to the fact that the sensor provided is only sensitive to the fetal kicks during this period. Therefore, it can be said that the sample utilized in this study represents the large population of pregnant mothers whose gestation age is between 26 weeks and 40+ weeks. The readings were taken in the duration from August 2019 to January 2020. No exclusions were made during the clinical testings. However, When conducting the analysis in this study only singleton pregnancies were considered. The age of the participants varied from 22 years old to 35 years old and the gestation age of fetuses varied from 26 weeks old to 40+weeks old.

During both these phases pregnant mother’s aural consent was obtained and written down prior to acquiring data and a thorough explanation on the process of acquiring data as well as the final target of the research was given. Then basic details about the mother as well as the fetus were recorded. The data collected were, mother’s age, fetal age, fetal gender, number of previous pregnancies, expected delivery method and additional comments. After that the belt containing the sensor was placed around the mother’s abdomen and secured to reduce the movements of the belt relative to the mother’s abdomen. Then the mother was advised to stay in a comfortable position and advised to refrain from making large movements. In phase one most mothers chose to lay down while a few were sitting up. However, during phase two every mother had to lay down in order to accommodate the ultrasound probe as well as the device. Each session was approximately 20 minutes long and for every mother, a single session was conducted. Therefore, more than 5 hours of readings were acquired.

#### Class identification

In previous studies identification of fetal movement was considered to be a two-class problem. The most common method of defining classes were fetal movements and mother’s respiratory movements. However, when observing the data gathered during the clinical testing, it was observed within a session a mother may introduce different kinds of artifacts such as cough, hiccup and laugh. However, when observing the time domain signals of each of these artifacts it was observed that the acceleration variation of maternal laugh is the most similar to the acceleration variation of a fetal movement. Moreover, maternal laugh is the most frequent artifacts that occurred during the clinical testing period. Hence, there’s a high probability that the algorithm may identify a maternal laugh as a fetal movement which will, in turn, result in a false positive result which is the most undesirable case in this application. Therefore, to improve the true positive rate, as well as to reduce the false positive rate, maternal laugh is also taken into consideration as a separate class. Therefore, during phase one three classes were introduced. They were fetal movement, maternal laugh and mothers respiratory movements. During phase two, three types of fetal movements were observed. They are limb movements, rotations and whole body movements. During the second phase, it was observed that fetal limb movements were the most prominent movements felt by mothers and most of the time these were the type of movements identified as fetal movements by mothers. Furthermore, fetal limb movements were easy to be detected with ultrasound and the whole-body movements were slow and infrequent during the window of observation for most of the subjects. Moreover, in a study conducted to observe the maternal perception of fetal motor activity it was observed that there’s a higher probability mothers may identify fetal limb movements as fetal movements than any other type of fetal movement [20]. Therefore, only fetal limb movements were considered as fetal movements in the second phase of clinical trials. Therefore, the three classes identified during phase two are fetal limb movements, maternal laugh and mother’s respiratory movements. The frequency of each class occurrence can be observed in Table 1.

**Table 1:** Number of occurrences of the three classes with gestation age.

FetalAge(Weeks)	Class 1	Class 2	Class 3
**27–31**	174	35	263
**32–35**	265	78	360
**36–40+**	583	163	954
**Total**	**1022**	**276**	**1563**

The total number of occurrences of each class can be observed. The fetal movement realizations are classified as Class 1, Maternal laugh realizations are classified as Class 2 and Maternal respiratory movement realizations are classified as Class 3.

#### Product user feedback

The end goal of this research was to design and fabricate a fetal movement monitoring system which can be used by pregnant mothers at home. The user experience of the pregnant mother who is the test case is of importance as observed by us for the design and the fabrication of the device. Therefore, during the first phase of clinical testing, free response feedback was taken from the mothers. In it initially, a brief description of our proposed system was given. Then information on the current methods utilized by the mothers was taken. Finally, feedback on the proposed system as well as the device was taken. Following observations were made from the feedback received. Every mother interviewed kept track of fetal movement during the pregnancy as advised by their obstetrician. However, this was done by keeping track of whether a fetal movement occurred during each hour of the day. The general opinion of the mothers was that this method was unreliable and a nuisance. Furthermore, they were advised to observe fetal moments immediately after consuming food. On average, a mother with a healthy fetus undergoes three ultrasound scans during the gestational period and undergoes a Cardiotocography (CTG) scan daily when impatient at the hospital. More than 80% of the mothers interviewed had a favourable opinion about the proposed system of fetal movement monitoring, while approximately 15% were indecisive. Only less than 5% of mothers had an unfavourable opinion about the proposed system. Moreover, almost every mother interviewed reacted favourably to the idea of including a mobile application to the proposed system.

### Observations

**Fig 2:**
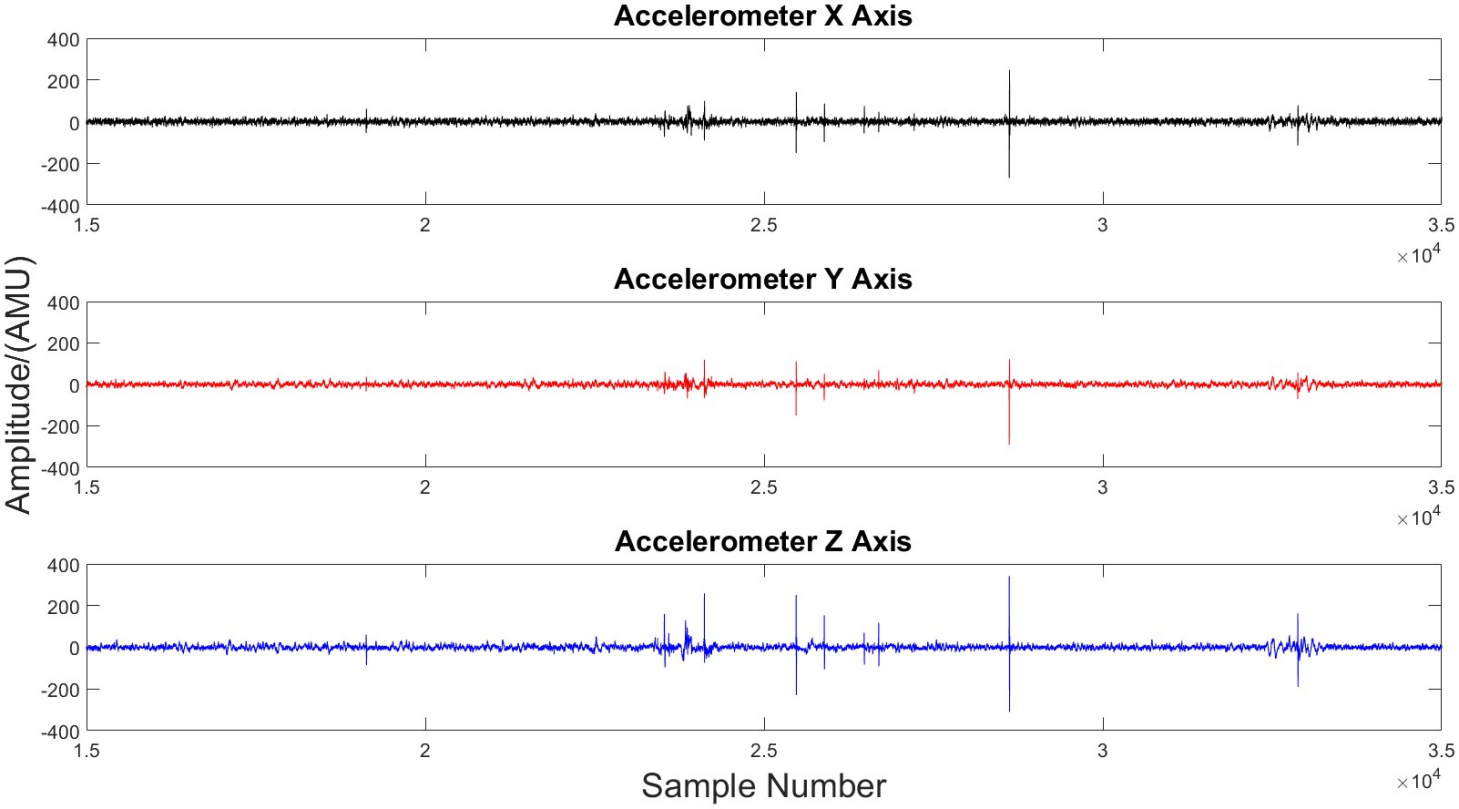
The accelerometric data along the three axes, X-Axis, Y-Axis and Z-Axis. The raw time domain data captured from the sensor are visualized in this figure. The acceleration recorded along the three axes can be observed separately. The acceleration recorded at each instance is plotted against the sample number.

The data obtained from the sensor were stored in the micro SD card which was later transferred and analysed. Prior to conducting analysis, several important observations were made. The accelerometer in the sensor measures the acceleration along the three axes: X-Axis, Y-Axis and Z-Axis. Z-Axis records the acceleration variation normal to the mother’s abdomen while X and Y axes record the acceleration variation along the plane of the abdomen. Raw time-domain data obtained can be observed in Fig 2.

**Fig 3:**
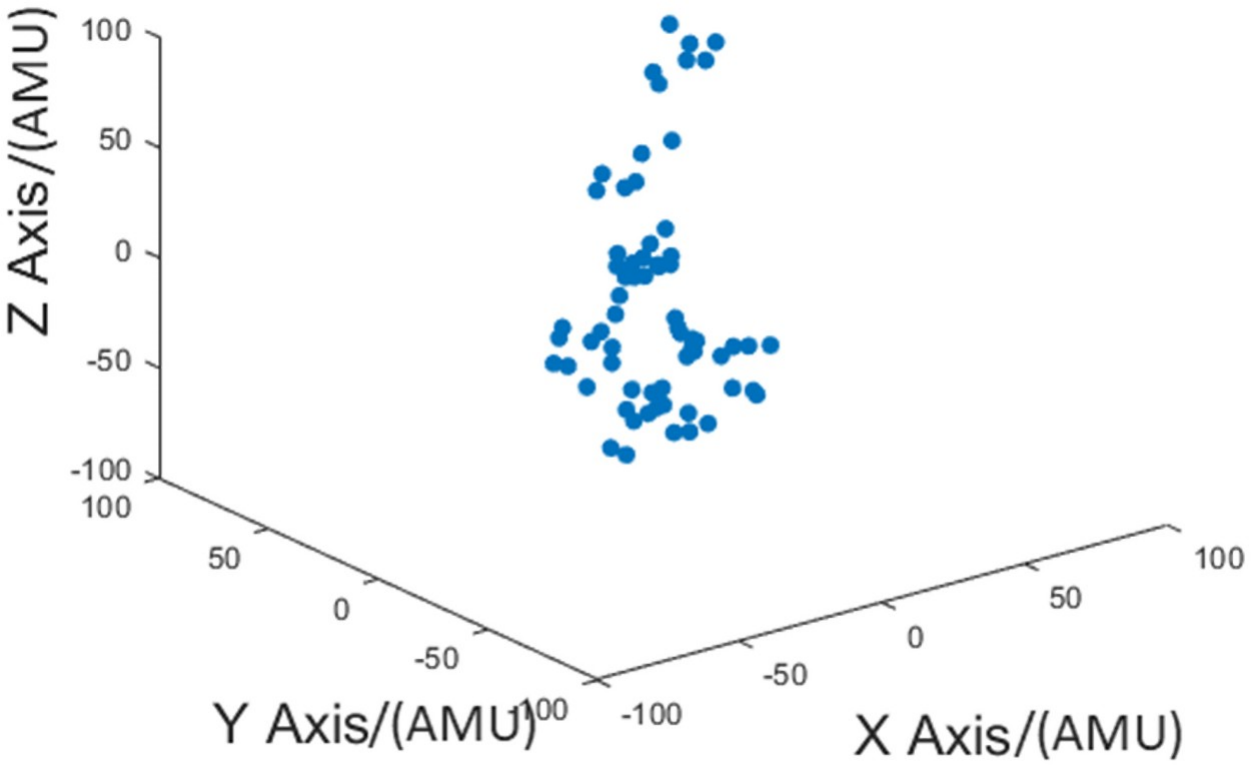
The variation of data point in the 3 dimensional space during a fetal movement realization. The raw time domain data captured from the sensor are visualized in this figure. Each point in the figure represents an instance a sample is taken. A single point is plotted such that the magnitude along each axis corresponds to the magnitude of acceleration in each direction at one instance. This figure was generated by plotting the data from a fetal movement realization. The span of each axes is set to a constant range of -100 to +100. Therefore, the difference in variation of acceleration along each axes can be observed clearly.

From Fig 2 it can be observed that the variation along the Z-Axis is more prominent than the variation along the other two axes. This argument can be further solidified by observing Fig 3. In Fig 3 the variation of data point in the 3 dimensional space can be observed during a fetal movement realization. In it, it can be observed that the most prominent variation is along the Z-Axis. Therefore, only Z-Axis data were utilized when conducting the analysis. Furthermore, this results in a reduction of computational capacity requirement, which in turn, is favourable when conducting complex algorithms.

**Fig 4:**
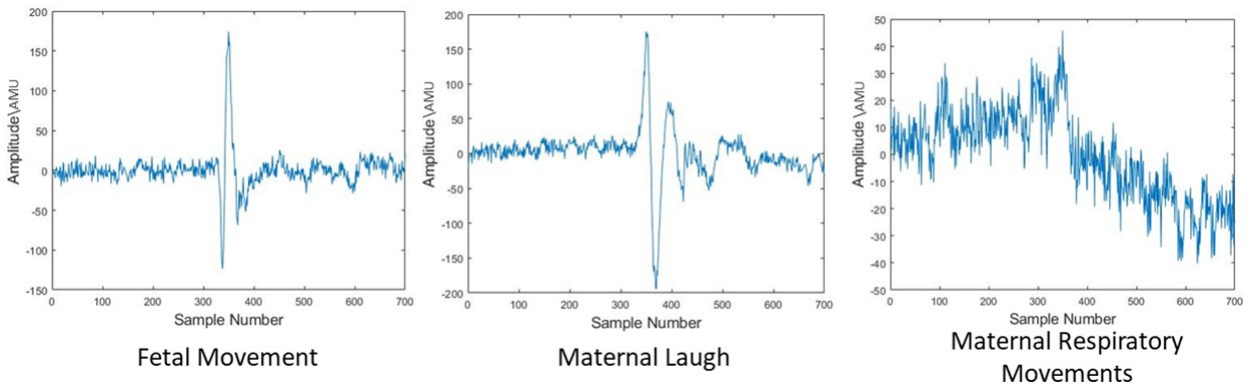
The accelerometric data of realizations from the three classes. The raw time domain data captured along the Z-Axis from the sensor are visualized in this figure. The first sub figure shows a realization of a fetal movement. The next sub figure shows a realization of a maternal laugh and the final sub figure shows a realization of a maternal respiratory movement. In all figures the recorded acceleration along the Z-Axis is plotted against the instance it is recorded.

During both phases of clinical testing, the realizations were segmented into three classes. They are fetal movement, maternal laugh and maternal respiratory movements. Visually the time-domain representation of the fetal movement and maternal laugh have similarities while clear discrimination can be made between these two and maternal respiratory movements. This can be observed in Fig 4.

**Fig 5:**
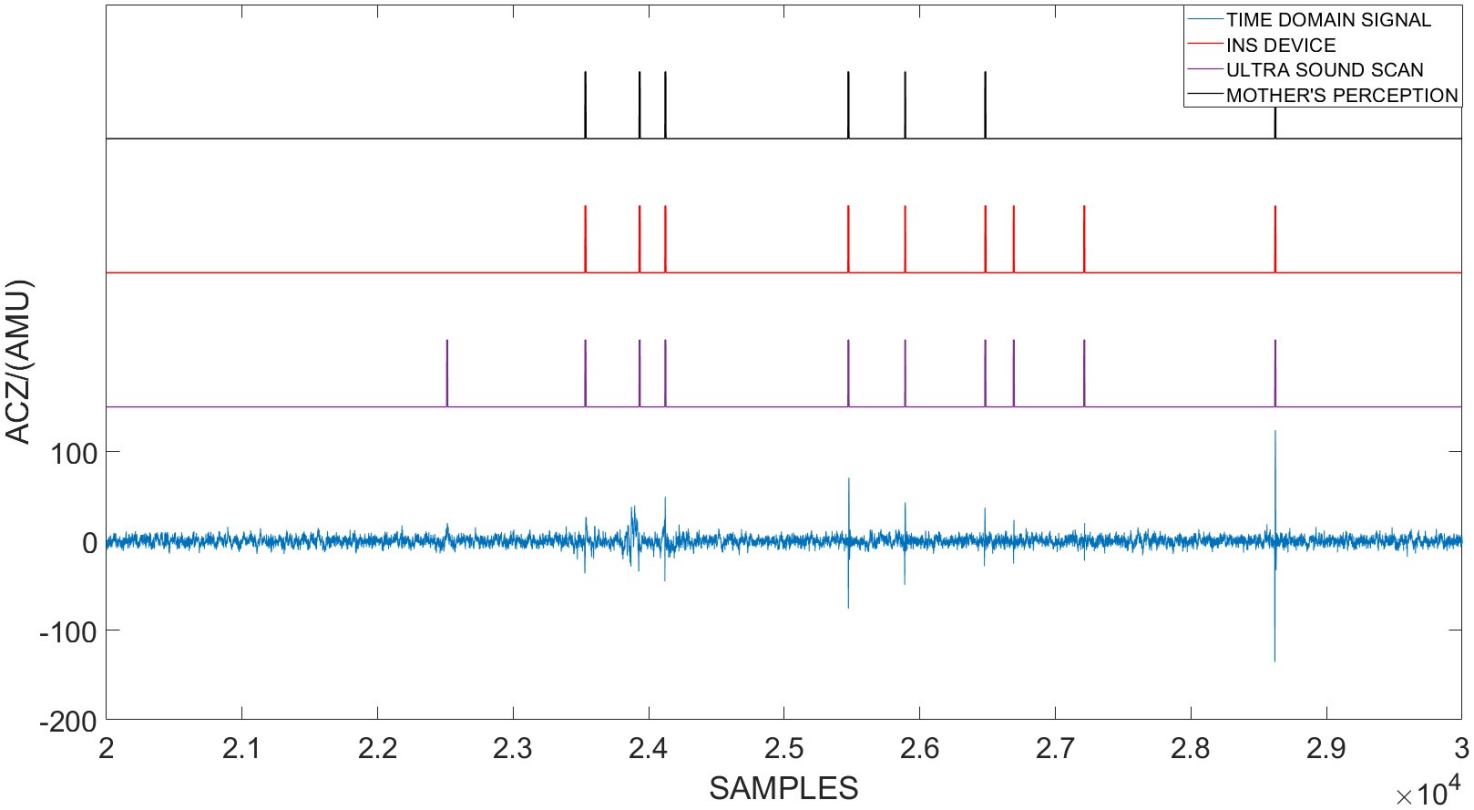
The time-domain signal of a singleton pregnancy observed by the sensor and the fetal movement identified by each method. In this figure the time domain accelerometric data along the Z-Axis recorded during a single session is plotted against the sample number. This session was conducted on a pregnant mother with a singleton pregnancy. During the session three methods were utilized to identify the occurrence of a fetal movement. They are: An abdominal Ultrasound scan, mother’s perception and the in-house fabricated device. The instances where each method identified the occurrence of a fetal movement are indicated by peaks.

**Fig 6:**
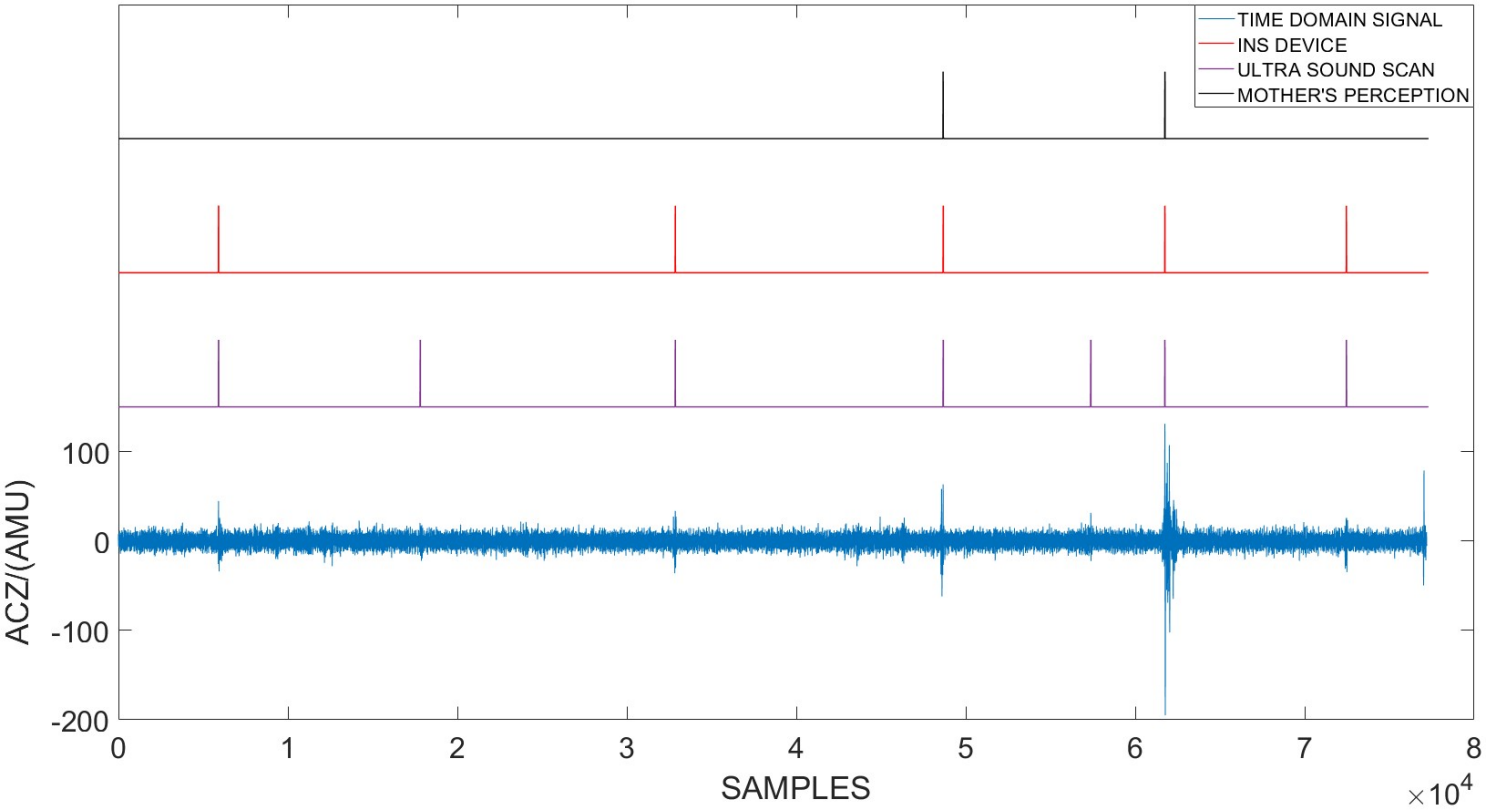
The time-domain signal of a singleton breached pregnancy observed by the sensor and the fetal movement identified by each method. In this figure the time domain accelerometric data along the Z-Axis recorded during a single session is plotted against the sample number. This session was conducted on a pregnant mother with a singleton breached pregnancy. During the session three methods were utilized to identify the occurrence of a fetal movement. They are: An abdominal Ultrasound scan, mother’s perception and the in-house fabricated device. The instances where each method identified the occurrence of a fetal movement are indicated by peaks.

During the second phase of the clinical testing procedure, an abdominal ultrasound was conducted to validate the results. The time-domain signal of a singleton pregnancy observed by the sensor and the fetal movement identified by each method can be observed in Fig 5. Furthermore, a similar diagram of the time domain signal of a breached fetal can be observed in Fig 6.

If the abdominal ultrasound is to be considered as the ground truth, it can be observed that some fetal movements were not felt by the mother as well as the device. It can be observed from the Fig 5 that in the normal singleton pregnancy most fetal movements identified by the ultrasound were also observed by the mother and the device. However, when the fetus is breached, most of the fetal movements identified by the ultrasound were not observed by the mother but they were observed by the device. Therefore, it can be derived that this proposed system can be utilized to identify and monitor fetal movement which can not be felt by the mother. A similar analysis was conducted for the entire data set obtained during the second phase. It was observed that 65.56% of realizations were identified by all three methods. However, 15.56% of realizations were identified by the ultrasound and the device and 18.89% of realizations were only identified by the ultrasound. The summery of this data can be seen in Table 2. Furthermore the mothers were not able to identify fetal movements which were not captured by the ultrasound scan and all the movements felt by the mothers were also captured by the device.

**Table 2:** Number of realizations identified and observed by each method during the second phase of clinical testing.

Ultra Sound	Device	Mother’s Response	Number of Detected Kicks	Total Percentage(%)
1	0	0	17	18.89
1	1	0	14	15.56
1	1	1	59	65.56

During the second phase of clinical testing three methods were utilized to identify the occurrence of a fetal movement. They are: the abdominal ultrasound, the in-house fabricated device and mother’s response. In the table ‘1’ indicates that the relevant method was able to identify the fetal movement and ‘0’ indicates that the method was not able to identify the fetal movement. For instance the first data row of the table indicated the number of realizations captured only by the abdominal ultrasound. The second row indicate the number of fetal movement captured by the ultra sound and the in-house fabricated device. And the final row indicate the fetal movements captured by all three methods.

### Signal analysis

Several combinations of signal processing algorithms were administered in order to obtain an optimum algorithm. The main objective of the algorithm was to successfully compute the number of fetal movement occurrences in a single session and to minimize the probability of false positives as these could result in extensive adversarial effects. The entire algorithm was implemented using MATLAB and later implemented on Android studio in order to implement this algorithm on a smart phone.

**Fig 7:**
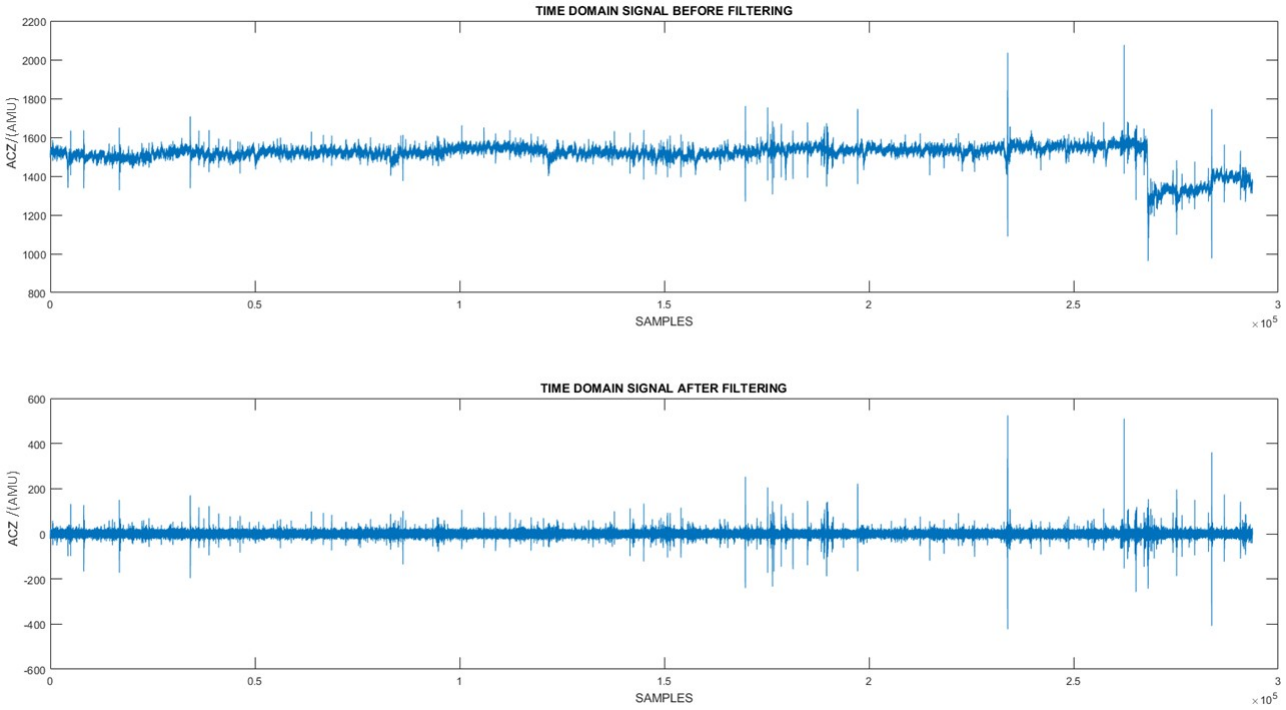
The raw time domain data captured from the sensor vs the time domain data when a custom high pass filter was applied. In this figure a comparison between the filtered and non filtered time domain data can be made. The Z-Axis acceleration data of a single session is used for the comparison. In the non filtered signal significant shifts in the time domain signal can be observed. However, they are eliminated when the high pass filter was implemented.

**Fig 8:**
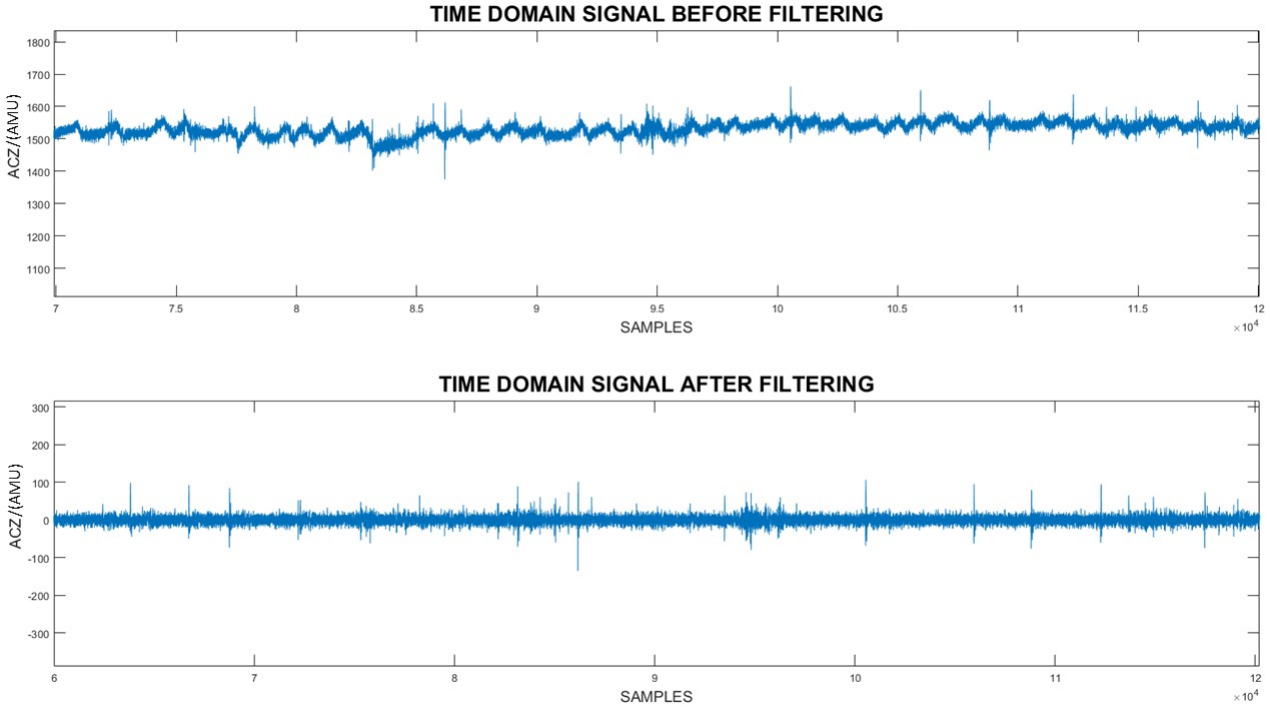
The raw time domain data captured from the sensor vs the time domain data when a custom high pass filter was applied (zoomed-in). In this figure a zoomed-in comparison between the filtered and non filtered time domain data can be made. Z-Axis accelerometric data from a segment of a session is used for the comparison. In the non filtered signal a sinusoidal pattern can be observed. It is due to the effect maternal breathing has on the sensor. However, as it can be observed this pattern was removed when the custom filter was implemented.

**Pre processing**. Initially the signals obtained were observed and it was noticed that due to maternal movements the signal has shifted as seen in Fig 7. Furthermore maternal breathing has introduced a periodic noise component which can also be observed in Fig 8. Even though, the mothers were advised to restrain from making large movements practically this is not the case [2]. Therefore, a simple method was utilized to filter out the noise due to maternal movement as well as due to breathing. Hence, the raw time domain signal was initially sent through a high pass filter which was utilized in a similar research study done previously [13]. As it can be observed in Fig 8 when the filter is applied maternal movements as well as maternal breathing noise were eliminated.

**Realization segmentation**. The output signal from the pre processor was then segmented in to realizations. These segments had a width of 200 samples. This sample size was selected by observing the average length of fetal movements and maternal laughs. The realizations were classified into three classes: fetal movement, maternal laugh and maternal respiratory movements. The window segmentation is done differently during the training and the application. During the clinical testing, maternal perception and the ultrasound were considered to be the ground truth of fetal movement occurrence. Therefore, when segmenting the data, the procedure followed is as follows. Initially, the instances where a fetal movement or maternal laugh have occurred were identified via the input of the button system. It was noticed that mothers tend to press the button a few seconds after the actual occurrence of a fetal movement. This was due to their response delay. Therefore, after identifying the instances the vicinity was checked for a peak and the sample was selected such that the peak is set as the median of the sample and sample width is set as 200 samples. These were then labelled accordingly. Samples from instances devoid of fetal movement and maternal laugh were selected as maternal respiratory movement realizations. However, during the application process realization segmentation was done differently in order to reduce the computational burden. In Fig 4 it can be observed during a fetal movement as well as maternal laugh the amplitude of the signal increases significantly. Therefore, when the program receives a time-domain signal initially an amplitude threshold was applied and peaks were identified. Then, realizations were segmented such that peaks are the median of the sample with a width set as 200 samples. These realizations were fed into the algorithm containing the pre-trained network.

**Short time fourier transform**. The raw accelerometric data are segmented into three classes: fetal movements, maternal respiratory movements and maternal laugh. Each signal category contains unique features. However, conducting only time domain analysis will inhibit the ability to extract these features in order to classify them in to each class. Therefore, it was required to visualize them in a more descriptive manner. Furthermore, the input for the subsequent algorithm is required to be two dimensional. To achieve this purpose, Short Time Fourier Transform (STFT) was utilized to generate Magnitude Spectrogram from the time domain accelerometric readings [21]. The Magnitude Spectrogram is one of the most expressive signal representation methods, because it represents the intensity plot of frequencies of a signal which varies with time [22].

**Non-negative matrix factorization**. During this study the final classification is to be done via a neural network. When implementing such networks the computational capacity plays a major role. Therefore, further data reduction methods were explored in order to reduce the computational burden on the final classifying neural network. In a previous study conducted Non-Negative Matrix Factorization (NNMF) and Spectral clustering algorithms were implemented on an accelerometric data set to discriminate and identify fetal movements [16]. In that study the two algorithms were compared in their ability to identify fetal movements from accelerometric data. It was concluded that NNMF Algorithm performs better and in turn it can be derived that it is a viable option to be utilized in this study as a data reduction method.

Non-Negative Matrix Factorization (NNMF) is widely used in signal processing to reduce the dimension [23]. Applications of NNMF range from simple text document clustering to advanced biological data mining [24, 25]. It is an advanced signal processing technique employed to identify and extract hidden features from raw signals. The input to the NNMF algorithm should be a non-negative matrix and the algorithm will generate two low rank non-negative matrices. This basic NNMF algorithm can be mathematically illustrated as follows [26].

$$\begin{array}{c} V\approx WH \end{array}$$ (1)

Where, *V*, *W*, *H* ≥ 0

In this study of fetal movements identification application, the magnitude spectrogram was utilized as the input non-negative matrix V. Following the NNMF algorithm two matrices are generated. They are the Basis Matrix W and the Activation Coefficient Matrix (Abundance Matrix) H. These two matrices are generated such that the Basis Matrix contains all the features of the given magnitude spectrogram while the Activation Coefficient Matrix contains the proportional factors of bases.

#### Convolutional neural network

From Fig 4 it can be observed that the captured signals are too noisy. Therefore, the possibility of successfully implementing a simple algorithm is very low. Moreover, several studies were conducted previously to the same data set utilizing simple algorithms such as eigen factorization and simple neural network [15, 27]. However, the accuracies reached by these algorithms were quite low. Furthermore, another study was conducted on the same data set and was able to attain higher accuracy [16]. However, the algorithm in that study was locally trained for individual mothers which is not practical. Convolutional Neural Network is one of the most common classification methods utilized in biomedical signal processing [28, 29]. Therefore, a CNN architecture was implemented in order to globally train the data. However, when implementing the CNN architecture a CNN with a minimum depth was selected to relax the computational burden.

In order to classify the three classes the output from the above algorithm was fed in to a Convolutional Neural Network. Due to the differences in frequency of occurrence of the three selected classes, unequal samples were fed into the CNN algorithm. The samples were separated into testing and training categories on the basis of this inter-class ratio. 80% of each class was utilized for training and the remaining 20% was utilized for testing. The selection process was random. The size of a realization fed into the CNN algorithm was 64x26 pixels and images were RGB. Following parameters of the CNN algorithm were varied and optimum values for each layer was obtained. The values of individual layers can be observed in Table 3. When training the network RelU Activation function was utilized and three fully connected layers were implemented for each class. Stochastic gradient descent method was used to train the network.

**Table 3:** Parameters of the three fully connected layers implemented in the convoluted neural network.

Parameter	Layer 1	Layer 2	Layer 3
**Neuron Size**	5 × 3	5 × 2	5 × 2
**Number of Neurons**	60	50	40
**Learning Rate**	0.0001	0.0001	0.0001
**Epochs**	80	150	300

In this table the parameters varied during implementing the convoluted neural network can be observed. The optimum parameters of each layer are mentioned.

### Mobile application

The final aim of this research study is to provide pregnant mothers with a wearable non-invasive device which can count and monitor fetal movement reliably. Therefore, initially a wearable deice was designed and fabricated. Then further studies were carried out to identify the best digital implementation method. During these, the digital literacy of the country was analysed. For this purpose, data released from the Department of Census and Statistics, Sri Lanka were utilized and following observations were made [30]. The overall computer literacy of the female population during the year 2019 was 28.3%. However, the average age group most pregnant mothers fall into is 20 to 35 years. The computer literacy of this age group vacillated between about 50%. Compared to computer literacy, digital literacy of females have a higher value which is around 40%. The digital literacy of females around the age of 20 to 35 vacillates between around 75%. Therefore, it can be concluded that more pregnant mothers in Sri Lanka have better digital literacy than computer literacy. Hence, the smart phone was chosen as the digital implementation method. Moreover, it was derived from the product user feedback data, that most mothers would prefer to use a mobile application along with the device.

**Fig 9:**
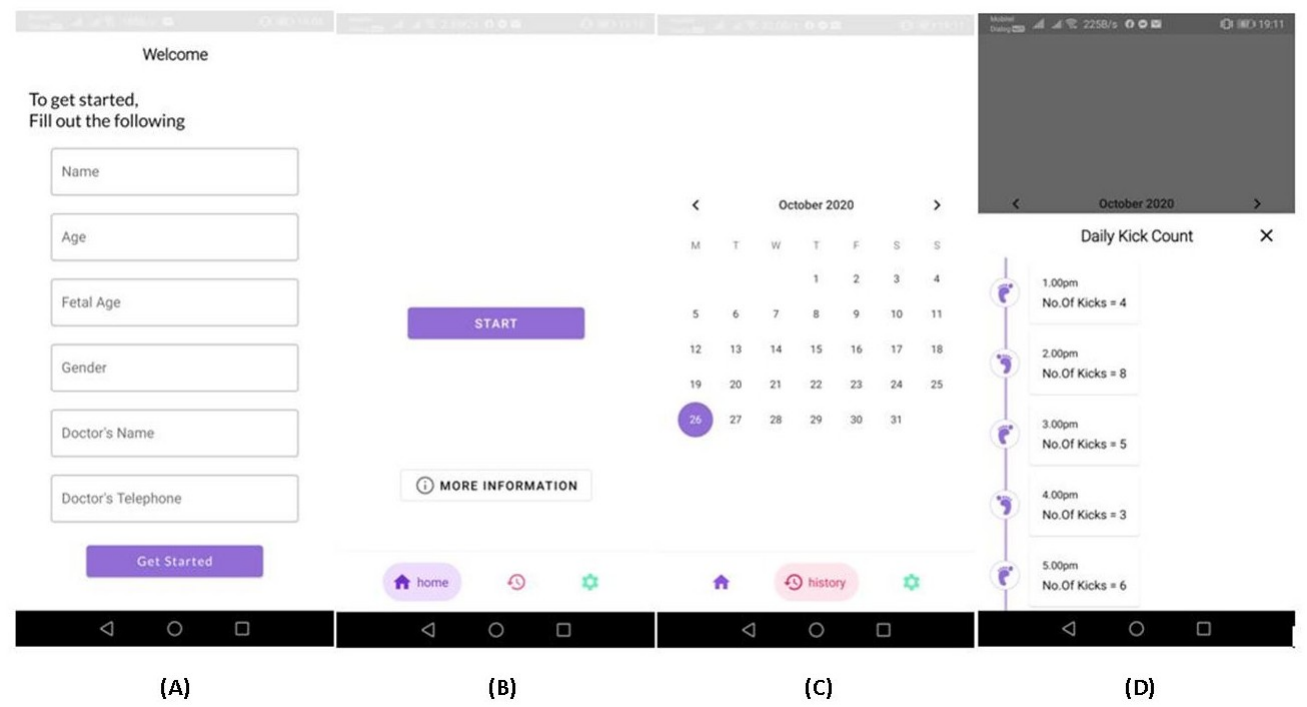
Designed mobile application interface. In this figure few screenshots captured from the designed mobile application can be observed. **(A)** This shows the interface when the app is launched initially. There details of the pregnant mother as well as the obstetrician are collected. **(B)** This shows the interface where a mother can upload the recorded data from the micro SD card. When the ‘Start’ button is pressed the data will be captured by the software and sent to the cloud to be analyzed. Subsequently the number of fetal movements occurred during the session will be displayed. **(C),(D)** As shown, the history of the recorded data can be also viewed.

Therefore, as the final system, the data capturing was done via the device in Fig 1. One of the main benefits of the proposed system is its non-transmitting quality. Hence an SD card is used to store the captured data from the device. Moreover, the main goal of the system is to monitor and identify an abnormality in fetal movement patterns in long term. Hence, real-time monitoring is not done in the proposed system. The mother has to manually remove the SD card from the device and insert it into the mobile phone in order to transfer data. This can be done either after each session or after several sessions. The app will fetch all the new sessions and give out the kick count of each individual session. Initially, although an attempt was made to conduct an analysis within the smartphone, it was not successful as common smartphones used by pregnant mothers lacked the computational capacity to run the algorithm. Furthermore, the size of the data file of a single session is compact. Therefore, as a solution to these problems the data is then transferred to an online cloud and the analysis was conducted remotely. At the end of the analysis the number of kicks recorded within the session will be sent back to the mother as well as to her obstetrician. When designing the mobile application the software Android Studios was utilized and when conducting the analysis remotely the Wamp Server Software was used. The mobile application was designed in a manner that is attractive as well as user friendly. The mobile application will store and record the fetal movement patterns as well. The user interface of the mobile application can be seen in Fig 9.

## Results

**Fig 10:**
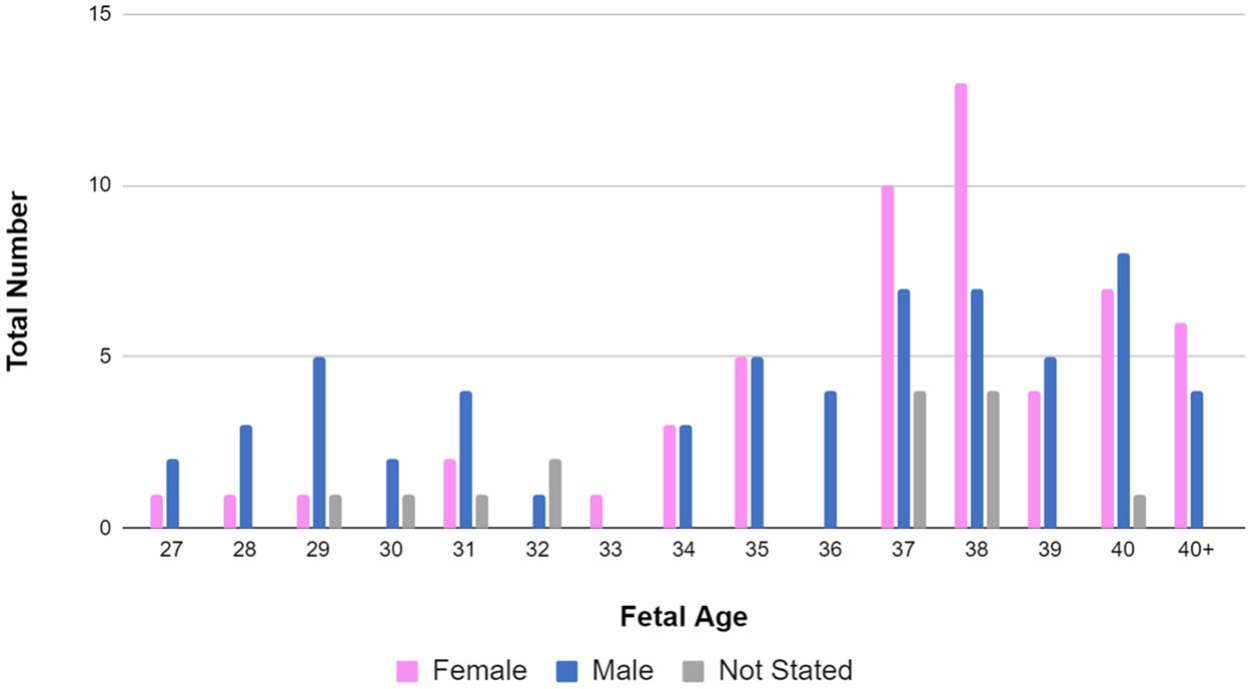
Distribution of gestational age and the fetal gender of the test group. The general distribution of fetal age of the test group can be observed in this figure. The distribution is distinguished based on the gender of the fetus. the height of each bar indicates the number of fetuses in that category. The gestation age of the test group varied from 27 weeks to 40+ weeks and a higher number of fetuses aged above 37 weeks can be observed.

During the clinical testing, readings from more than 120 mothers were taken. The distribution of gestational age and the fetal gender of the test group can be observed in Fig 10. From this figure, it can be observed that the gestation age of the test group varied from 27 weeks to 40+ weeks, where 40+ implies the fetus age is beyond 40 weeks old. Furthermore, it can be observed that the test group mostly contained fetuses whose gestation periods were beyond 37 weeks and the gender distribution was approximately uniform. However, the test group contained a higher number of younger male fetuses than female fetuses and a higher number of older female fetuses than male fetuses. There were few fetuses where the gender was not stated.

The algorithms stated above were implemented on this data set and the results were observed. With the aim of obtaining an optimum algorithm, several combinations of algorithms were implemented. They are:

1. **Algorithm 1**: Segmentation—STFT—CNN
2. **Algorithm 2**: High pass filter—segmentation—STFT—CNN
3. **Algorithm 3**: High pass filter—segmentation—STFT –NNMF(W)– CNN
4. **Algorithm 4**: High pass filter—segmentation—STFT –NNMF(H)– CNN

**Fig 11:**
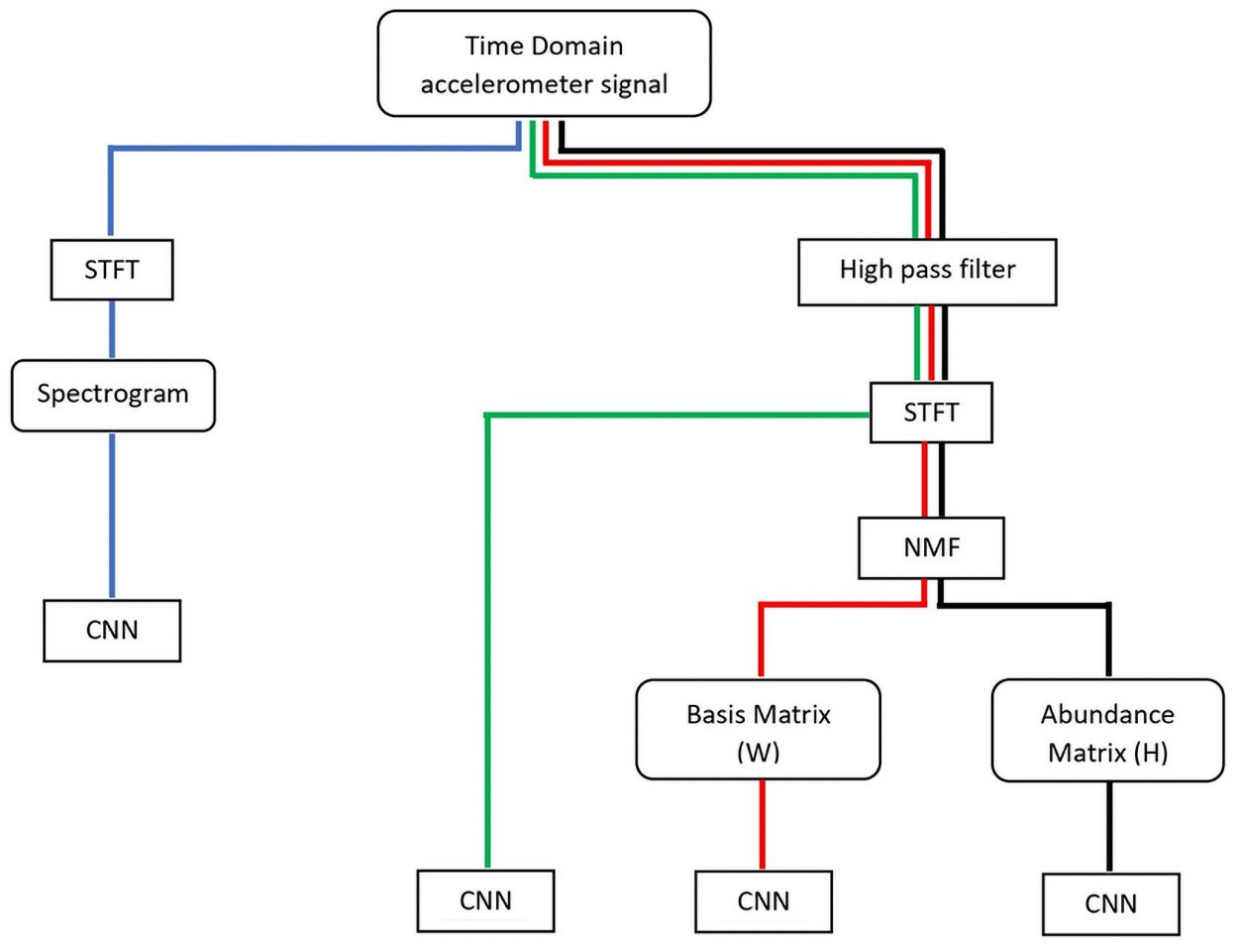
The flow of the four algorithms. The general flow of the four algorithms is presented in a graphical manner. The individual signal processing method utilized in each algorithm can be clearly observed.

These four algorithms were implemented on to the data set in order to identify the best algorithm to be utilized for the proposed system. The general flow of the four algorithms can be observed in Fig 11. Furthermore, by observing the difference in the performance of each algorithm the effect of each individual signal processing method used can also be understood. The effect of implementing a high pass filter as a pre-processing step can be observed by comparing the performance of algorithm 1 and algorithm 2. Short-Time Fourier Transform(STFT) is utilized in all four algorithms in order to represent the time domain data in a more descriptive manner which will, in turn, allow the neural network to identify time-frequency features, which may be hidden in the time domain data. However, since Spectrograms generated via STFT were greater in file size NNMF was utilized as a data reduction method, where the NNMF algorithm separated the STFT generated spectrogram into two distinct matrices. these two matrices were utilized in two algorithms, Algorithm 3 and Algorithm 4. The effect of this data reduction on the data set can be observed by comparing the performance of Algorithm 2 and Algorithm 3 as well as by comparing Algorithm 2 and Algorithm 4. Moreover, the performance of each matrix generated by NNMF can be compared by observing the results of Algorithm 3 and Algorithm 4.

**Fig 12:**
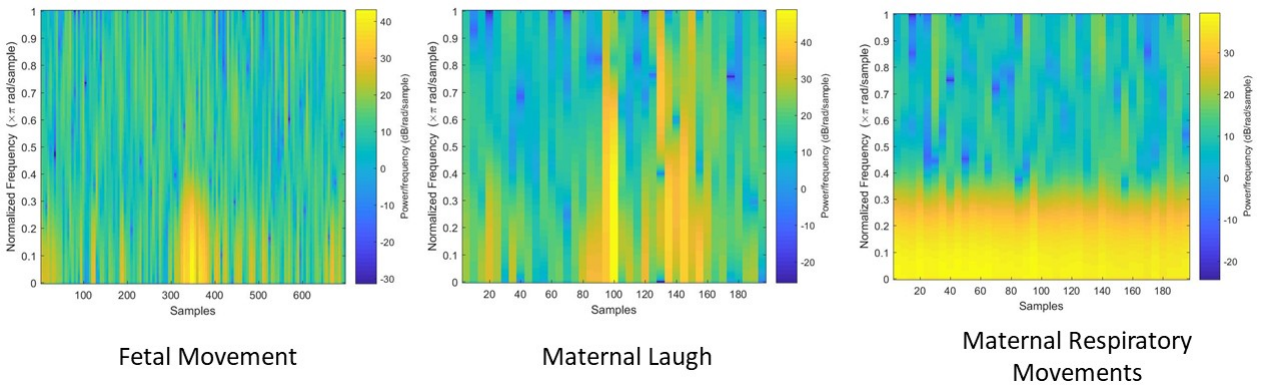
The resulting spectrograms of the three classes. The filtered time domain signal was fed in to a Short Time Fourier Transform (STFT) algorithm and the resulting figures are shown here. A figure of each type of realization can be observed and these figures are generated based on the output matrix of the STFT algorithm. The amplitude of the output matrix was utilized and the different colours corresponds to the amplitude level of each frequency at each instance.

In each algorithm immediately after the segmentation a short time Fourier transform was implemented. The resulting spectrograms can be seen in Fig 12. It can be observed that the spectrogram of the maternal respiratory movements is concentrated on to the lower bands of frequency, and this is constant through out the samples. However in the spectrogram of fetal movements this concentration occurs only in a smaller range of samples. Furthermore, the spectrogram of the maternal laugh signal differ from the other two as well. Therefore, it can be stated that implementing a standard short time Fourier transform can aid the discrimination process.

**Fig 13:**
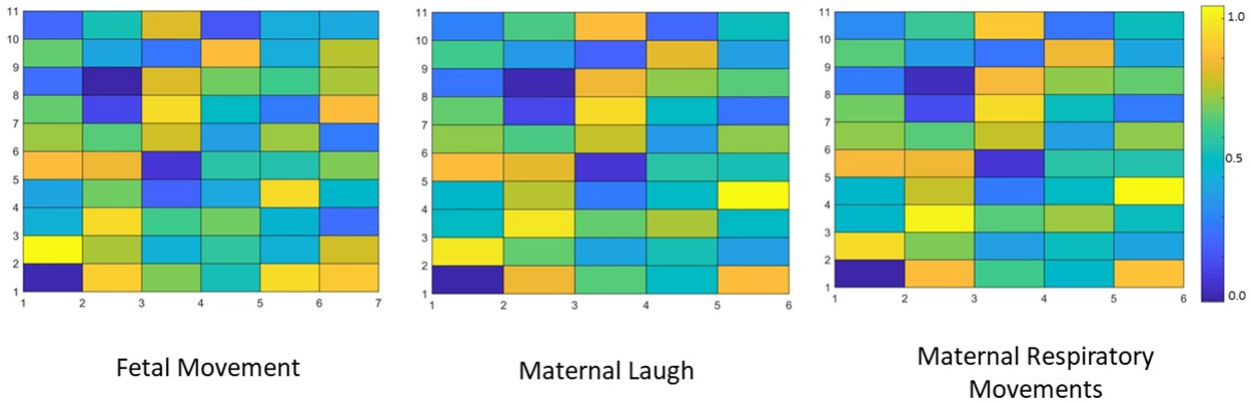
The resulting base matrices (W matrices) for three classes. The resulting base matrix(W matrix) of three different classes can be observed in this figure. The output of the NNMF algorithm are two matrices, W matrix and H matrix. In order to visualize the matrix the amplitude of each element of the matrix is converted in to a corresponding colour. As a result of this a figure in a grid format was obtained.

**Fig 14:**
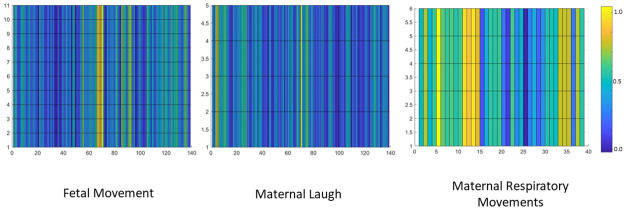
The resulting abundance matrices (H matrices) for three classes. The resulting abundance matrix(H matrix) of three different classes can be observed in this figure. Similar to what is done with the W matrix in order to visualize the matrix the amplitude of each element of the matrix is converted in to a corresponding colour. As a result of this a figure in a grid format was obtained.

Then a standard Nonnegative Matrix Factorization algorithm was implemented on to the spectrogram images. This factorized the spectrogram image into two images: the abundance matrix and the base matrix. As stated in the previous section the abundance matrix is identified as the H matrix and the base matrix is identified as the W matrix for easy reference. Then the matrices are visualized in to figures with a grid format. Similar to the figures generated by the STFT algorithm, the amplitude of elements of the output matrices were converted in to colours and visualized. The generated figures for the W matrix of three different realizations can be observed in Fig 13 and the generated figures for the H matrix of the same three realizations can be observed in Fig 14.

**Fig 15:**
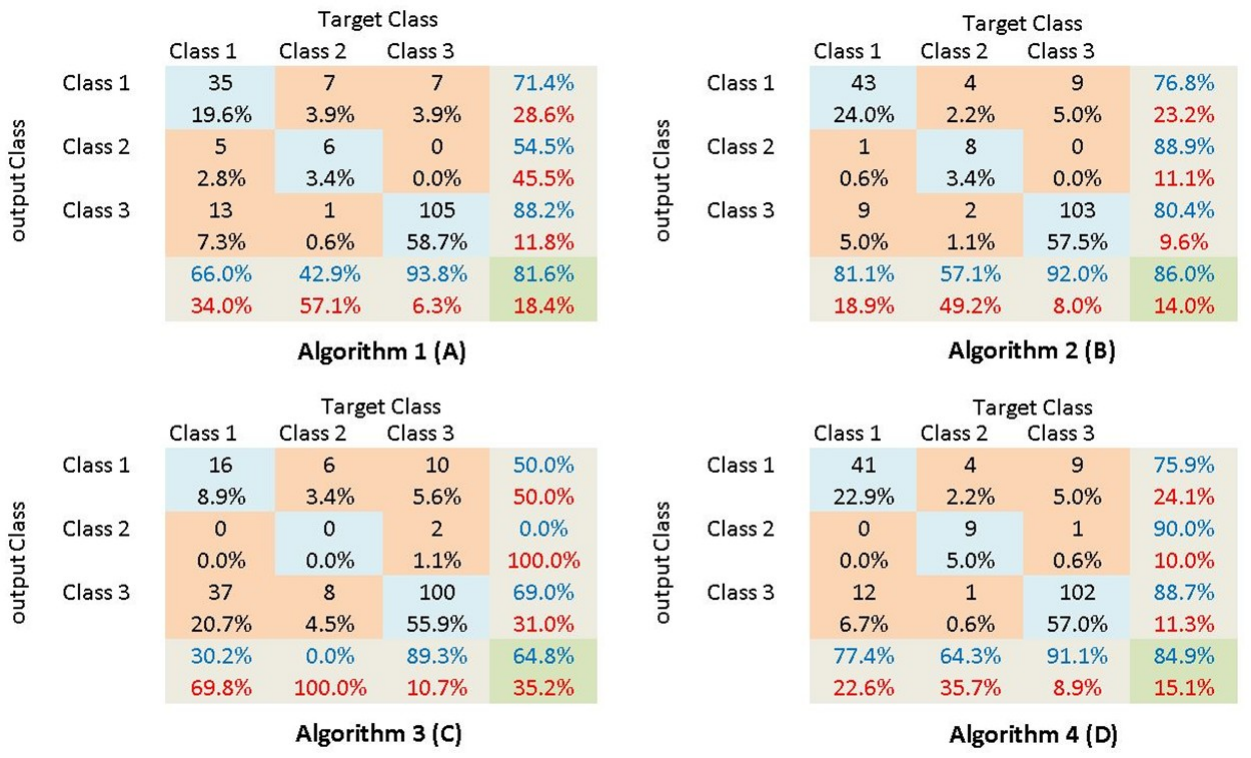
The confusion matrices generated for each algorithm. The confusion matrices obtained by implementing the four signal processing algorithms can be observed.

Then the spectrograms, and the base matrices and the abundance matrices were fed in to a standard convoluted neural network algorithm described above. From each following confusion matrices were obtained. In the given matrices Class 1 represents the fetal movement realizations, Class 2 represents the maternal laugh realizations and Class 3 represents the maternal respiratory movement realizations. And confusion matrices obtained by implementing the four algorithms are shown in Fig 15.

## Discussion

From the given confusion matrices following observations can be made. The confusion matrix in Fig 15(A) is obtained by implementing Algorithm 1, where the raw time-domain signal is fed into the STFT algorithm. From it, it can be observed while the accuracy of the algorithm is not at the best reasonable discrimination among classes can be obtained. The confusion matrix in Fig 15(B) is obtained by implementing Algorithm 2. In this initially, a high-pass filter was implemented to remove large maternal movements as well as maternal breathing pattern from the signal. Then the filtered signal was fed into the STFT algorithm and the remaining process is similar to Algorithm 1. By comparing the results obtained by implementing these two algorithms the effect of implementing a high-pass filter can be clearly observed. When comparing the two confusion matrices. it can be clearly observed that the true positive accuracy of Algorithm 2 is higher than the True positive accuracy of Algorithm 1. However, the most important observation is the reduction of the false-positive rate with the implementation of the high pass filter. In this application of fetal movement monitoring, higher attention is paid to the false positive rate. When a false positive occurs the observer will identify a fetal movement when actually a fetal movement has not occurred. Therefore, if the false positive rate is higher then the application will indicate a higher fetal movement rate than the actual. Therefore, mothers and health care providers won’t be able to identify a significant reduction in fetal movement rates early, which may lead to dire consequences. It can be observed that in Algorithm 2 the false positive rate is comparatively low than the false positive rate of Algorithm 1. From these observations, it can be observed that the introduction of the high-pass filter as a pre-processing step has improved the results of the algorithm.

The confusion matrices obtained by implementing Algorithm 3 and Algorithm 4 can be observed in Fig 15(C) and 15(D) respectively. In Algorithm 2 the entire spectrogram was fed into the CNN algorithm. However, in Algorithm 3 and 4, the spectrogram was factorized using the Non-Negative Matrix factorization and the resulting abundance matrices and the base matrices are fed in the CNN respectively. The main reason to factorize the spectrogram is to reduce the computational capacity required to run the CNN algorithm. However, the following observations were made by analyzing the confusion matrices. When considering the observations made when Algorithm 3 is implemented it can be noted that the performance of this algorithm is weak. The true positive rate is at the lowest of 65% and the false positive rate is at it’s highest of 9%.

However, the best results were obtained when the spectrograms are fed into the CNN rather than any factorized matrices. The true positive rate of Algorithm 2 is 86% and the false positive rate is around 7%. The next best result is observed when the (Abundance Matrix) H is fed into the CNN algorithm. The true positive rate of it is approximately 85% and the false positive rate is around 7.2%. When comparing these two algorithms the decrease in the true positive rate can be neglected. However, the false-positive rate will increase due to factorization. For the same explanation made above, in this application, more attention needs to be paid to the effect of the false-positive rate.

The main reason for the better performance of Algorithm 2 could be due to the properties of the factorization algorithm. When the spectrogram matrix is factorized into the abundance matrix and the base matrix the abundance matrix contains mainly the data pertaining to individual mothers. Therefore, when the abundance matrices are fed into the CNN algorithm since the algorithm is trained utilizing data obtained from several mothers the performance is low. However, if we were to train the network using the abundance matrices of an individual mother the performance may be better. This can be observed in Table 4 where Algorithm 2 to 4 are implemented on 6 individual mothers and the true positive rates are compared. In the table, it can be observed that for some mothers the true positive rate obtained using Algorithm 4 is higher. This solidifies the argument made above. However, training a network utilizing an individual mother is not feasible for the intended application. Furthermore, if the training is to be done to individual mothers a large number of samples need to be collected from individual mothers. This does not go along with the target of devising a universal system to monitor fetal movements. Furthermore, it can be observed the performance of Algorithm 3 is weak even if it was implemented on individual mothers.

**Table 4:** True positive rate of Algorithm 2 to 4 on individual mothers.

MotherIndex	A2 (%)	A4(%)	A3(%)
**1**	93.55	83.87	41.94
**2**	88.89	88.89	63.49
**3**	78.57	71.43	64.24
**4**	71.43	71.43	42.86
**5**	71.43	71.43	57.14
**6**	68.75	75.00	43.75

In this table the true positive rates observed when the three algorithms are implemented on six individual mothers. ‘A2’ refers to Algorithm 2, ‘A4’ refers to Algorithm 4 and ‘A3’ refers to Algorithm 3. The results of Algorithm 2 and Algorithm 4 are juxtaposed for the purpose of better discrimination among the two. The results from Algorithm 3 are also included.

The main goal of developing this system is to come up with a method to monitor and identify abnormalities in fetal movement patterns in the long term in a non-clinical setting. The existing methods for this are maternal perception and ultrasound scanning. Ultrasound scanning can only be conducted in a clinical environment and the repercussions of long term exposure to ultrasound scanning are not yet explored. Furthermore, maternal perception is unreliable as well as time-consuming for the mother. This proposed system will act as a substitute for the maternal perception of fetal movement monitoring. Hence, it can be concluded that the proposed system be implemented utilizing the best performing algorithm, which is Algorithm 2. This Algorithm provides a true positive rate of 86% while not ideal for clinical diagnosis is more than viable for an automated monitoring tool to detect whether there is cause for concern. If so it will provide sufficient information that will entice the mother to seek medical assistance under such a situation.

## Conclusion

The need for a proper system to monitor fetal movements in a non-clinical setup is of paramount importance in order to maintain fetal well-being. Although some previous research studies were conducted on this front, several crucial and novel concepts were introduced in this study. A complete system was introduced to be used by pregnant mothers to monitor fetal movement count in a non-clinical setting. While a significant amount of effort was spent on developing the algorithm as well as the sensing system an equal amount of effort was invested in designing and implementing proper ergonomics and a user-friendly interface to the system. It was made sure that the proposed system is feasible to be implemented. This was done by studying the preferences and habits of pregnant mothers. Furthermore, it was ensured that the system is user friendly in nature. This was aided by the extensive amount of surveys conducted while clinical testing procedures.

In the initial phases of the research, a proper non-invasive device was designed and fabricated. Then from the feedback received from pregnant mothers, it was further modified. Subsequently, a mobile application was developed to be used by mothers. Finally, four different algorithms were implemented on the data set to identify the most acceptable algorithm. From the results, it can be observed that out of the four algorithms while algorithm 1,2 and 4 performed to a satisfactory level the performance of Algorithm 3’s performance was poor. Hence it can be concluded that when the STFT generated spectrogram was factorized using NNMF most of the features that help to discriminate fetal movements from other artifacts are factorized into the abundance matrix(H) rather than into the base matrix(W). Hence the performance of Algorithm 3 was low. From the remaining algorithms, the true positive rate of Algorithm 1 is less than the other two (Algorithm 2 and 4). Furthermore, Algorithm 1’s false positive rate is comparably high. By comparing these results of Algorithm 1 with Algorithm 2 and 4 it can be observed that application of the high pass filter in Algorithm 2 and 4 result in a significant reduction of the false positive rate which is highly desirable. Furthermore, when comparing the performance of Algorithm 2 and Algorithm 4 when they are globally trained it was observed that Algorithm 2 results in a higher true positive rate as well as a lower false-positive rate, which is highly desirable. However as it can be observed in Table—when these two algorithms are trained locally the data reduced Algorithm 4 performs better than Algorithm 2. Hence followings can be concluded by comparing the performance of the four algorithms. Implementing a high pass filter as a preprocessing step results in the improvement of overall performance. Moreover, while in a globally trained environment the CNN architecture which is fed the spectrograms as the input performs better, whereas in a locally trained environment the CNN architecture which is fed the abundance matrix (H) of the NNMF decomposition performs better. Since in this application the CNN architecture is required to be trained globally Algorithm 2 was selected as the preferred algorithm.

## Supporting information

S1 File The code for the mobile application. The main fiction of this mobile application is to collect data from the SD card and to transfer the data to an online cloud. The analysis will be conducted in the cloud it self and the application will retrieve the kick count per session. Furthermore, it will store and record the fetal movement patters with date and time stamp.
(ZIP)
