# Supplementary material for: Novel non-invasive in-house fabricated wearable system with a hybrid algorithm for fetal movement recognition
Source: PLoS One. 2021 Jul 13;16(7):e0254560. doi: 10.1371/journal.pone.0254560 (PMC8277045; doi:10.1371/journal.pone.0254560)
Supplement: S1 File — The main fiction of this mobile application is to collect data from the SD card and to transfer the data to an online cloud. The analysis will be conducted in the cloud it self and the application will retrieve the kick count per session. Furthermore, it will store and record the fetal movement patters with date and time stamp. (ZIP) [file pone.0254560.s001.zip › KickCount/app/build/reports/lint-results-release-fatal.html]

Lint Report


Lint Report: No errors or warnings


Check performed at Fri Jul 24 21:56:53 IST 2020

Issue Types

*dashboard*Overview

## No Issues Found

Congratulations!
